# First data on the organization of the nervous system in juveniles of *Novocrania anomala* (Brachiopoda, Craniiformea)

**DOI:** 10.1038/s41598-020-66014-9

**Published:** 2020-06-09

**Authors:** Elena N. Temereva

**Affiliations:** 1 Moscow State University, Biological Faculty, Dept. Invertebrate Zoology, 119991 Moscow, Russia; 2 Faculty Biology and Biotechnology, National Research University Higher School of Economics, Moscow, Russia

**Keywords:** Developmental biology, Evolution, Neuroscience, Zoology

## Abstract

The organization and development of the nervous system are traditionally used for phylogenetic analysis and may be useful for clarification of evolution and phylogeny of some poor studied groups. One of these groups is brachiopods: most data on their nervous system organization were obtained in 19^th^ century. In this research, antibody staining and confocal laser scanning microscopy were used to study the nervous system of early ontogenetic stages of the brachiopod *Novocrania anomala*. Although *N. anomala* adults are thought to lack a supraenteric ganglion, a large supraenteric ganglion exists in *N. anomala* juveniles with either a trocholophe or a schizolophe. During ontogenesis, the supraenteric ganglion in the juvenile changes its shape: the commissure between the two lobes of the ganglion extends. This commissure possibly gives rise to the main brachial nerve in adults. The supraenteric ganglion gives rise to the cross (transversal) nerves that extend to the accessory brachial nerve, which gives rise to the tentacular nerves. In juveniles with a trocholophe, the accessory brachial nerve gives rise to the frontal and intertentacular nerves of tentacles that form a single row. When the trocholophe transforms into the schizolophe, the second row of tentacles appears and the innervation of the tentacles changes. The intertentacular nerves disappear and the second accessory nerve forms and gives rise to the laterofrontal tentacular nerves of the inner and outer tentacles and to the abfrontal nerves of the inner tentacles. The so-called subenteric ganglion, which was described as a ganglion in *N. anomala* adults, is represented by a large circumvisceral nerve in *N. anomala* juveniles.The results suggest that ‘phoronid-like’ non-specialized tentacles may be regarded as the ancestral type of tentacles for brachiopods and probably for all lophophorates. The presence of intertentacular nerves is the ancestral feature of all lophophorates. The transformation of the juvenile supraenteric ganglion into the main brachial nerve of *N. anomala* adults suggests that research is needed on the development and organization of the supraenteric ganglion and the main brachial nerve in other brachiopods, whose adults have a prominent supraenteric ganglion.

## Introduction

Brachiopods are marine benthic animals with sessile life styles. They are filter feeders, i.e., they use a specialized tentacular structure called the lophophore to capture food particles. Today about 400 extant species species are described, however more than 30,000 extinct species are known to science^1–3^. An understanding of the biology of the many extinct brachiopods requires research on the development and organization of all organ systems of extant species from different groups.

Although the organization of the nervous system is traditionally regarded as important for comparative anatomy and phylogeny, the anatomy of the nervous system of extant brachiopods has not been adequately studied by transmission electron microscopy, antibody staining, and confocal laser scanning microscopy. Most of the detailed morphological information we have today is gained by histological methods from the 18th century^4–7^. In recent studies, the phylum Brachiopoda is subdivided into three subphyla, whose relationships are still vague: Cranoiiformea, Linguliformea, and Rhinchonelliformea^8–10^. Transmission electron microscopy, antibody staining, and confocal laser scanning microscopy have been used, however, to obtain data concerning the lophophore, the nervous system of species in the subphyla Linguliformea^11^ and Rhinchonelliformea^12^. The nervous system of Craniiformea is still not studied by modern immunohistological methods.

According to some researchers, the metamorphosis of craniiform larvae may reflect some steps of brachiopod evolution^13–15^. The development of the nervous system was described in advanced larvae and juveniles of the craniiform species, *Novocrania anomala*^16^. According to the latter study, 17-day-old juveniles lack a large nerve center, and their nervous system is represented by several main nerve cords. This result is supported by previous study, according to which adults of *N. anomala* lack a supraenteric ganglion and possess only a subenteric ganglion^6^. At the same time, Altenburger and Wanninger^16^ neither described any nerve tracts related to the tentacular innervation nor any tentacular neurites in juveniles. The latter findings seem odd, because they indicate that juveniles lack tentacles (or innervation of tentacles) and do not feed for a prolonged period.

In all brachiopods, the body is surrounded by a shell consisting of a ventral and a dorsal valve^17,18^. Many brachiopods have a pedicle, which is attached to the substratum and which facilitates the movement of the whole body in response to local water currents. *N. anomala* lacks a pedicle; the ventral valve tightly adheres to the substratum^19^. The dorsal valve is mitriform. The organization of the *N. anomala* shell and body causes the tentacles to be near the substratum, which presents a potential challenge for the filtration of particles from the water column. This potential challenge suggests that the organization of tentacles and the lophophore in *N. anomala* might be different in other brachiopods.

The study of the nervous system and innervation of the tentacular apparatus is useful for the comparative analysis of the organization of different types of lophophores in brachiopods in particular and in lophophorates in general^20,21^. It is also important for the reconstruction of the ancestral type of lophophore and tentacles in the lophophorates. The first goal of this report is a detailed description of the nervous system in general and the innervation of the lophophore in particular in juveniles of *Novocrania anomala*. In this report, I also consider how these new data provide insight into the evolution of the tentacles and the lophophore in brachiopods and lophophorates in general.

## Results

### General morphology of the body and lophophore in juveniles

**Figure 1:**
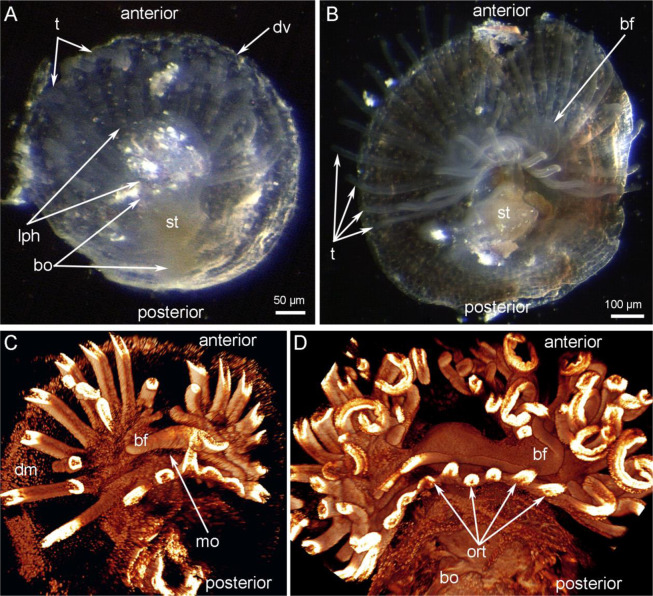
General morphology of juveniles of *Novocrania anomala*.(**A**) Photograph of a live animal with a trocholophe viewed from the ventral side; the lophophore is screened by the ventral mantle, (**B**) photograph of a live animal with a schizolophe viewed from the ventral side, (**C**) volume rendering of the whole body of a juvenile with a trocholophe (DAPI), (**D**) volume rendering of lophophore and part of the body of a juvenile with a schizolophe (DAPI). Abbreviations: bf, brachial fold; bo, body; dm, dorsal mantle; dv, dorsal valve; lph, lophophore; mo, mouth; ort, oral tentacles; st, stomach; t, tentacle.

Juveniles of *N. anomala* examined in this study had a dorsal valve that ranged from 0.5 to 1.0 mm in diameter; an increase in diameter presumably indicates a shift to the next ontogenetic stage (Fig. 1A,B). Most of the body occupies the posterior portion of the dorsal valve, but the tentacles extend anteriorly and occupy the mantle cavity. At early ontogenetic stages, juveniles have a trocholophe (i.e., a simple type of lophophore), which bears one row of tentacles that surround the mouth from the posterior side (Fig. 1C). In these early juveniles, a short brachial fold covers the mouth from the anterior side. At late ontogenetic stages, juveniles develop a schizolophe, a type of lophophore with two rows of tentacles (inner and outer) and a large brachial fold (Fig. 1D).

**Figure 2:**
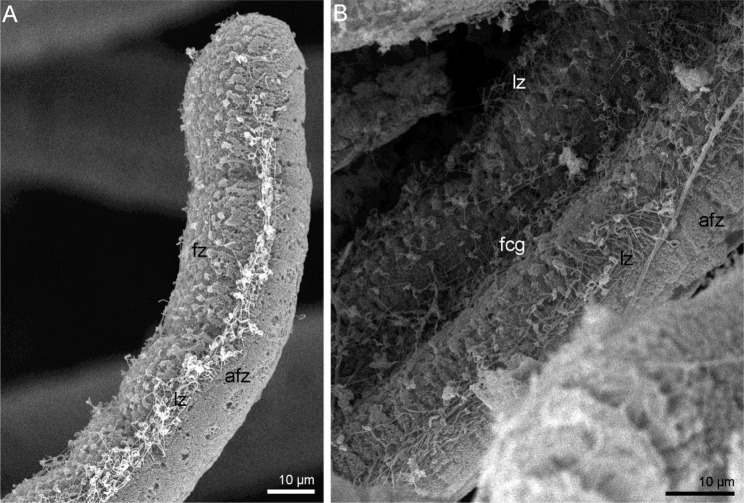
Organization of tentacles in juveniles of *Novocrania anomala*, SEM. (**A**) Tip of a tentacle of a juvenile with a trocholophe (i.e., a non-specialized “phoronid-like” tentacle), (**B**) a portion of the outer tentacle of a juvenile with a schizolophe. Abbreviations: afz, abfrontal zone; fcg, frontal ciliated groove; fz, frontal zone; lz, lateral zone.

Morphology of tentacles of a single row in the trocholophe of *N. anomala*, is similar. Each tentacle has a highly ciliated frontal surface that faces the mouth, heavily ciliated lateral zones, and a sparsely ciliated abfrontal surface that is opposite to the frontal surface (Fig. 2A). The two rows of tentacles in the schizolophe are formed by alternating inner and outer tentacles that differ in morphology. The inner tentacles are located near the brachial fold, and their morphology is similar to that of the tentacles in the trocholophe. The outer tentacles are located between the inner tentacles in a staggered order. The frontal surface of the outer tentacles forms a deeply ciliated groove (Fig. 2B).

### Anatomy of the nervous system

**Figure 3:**
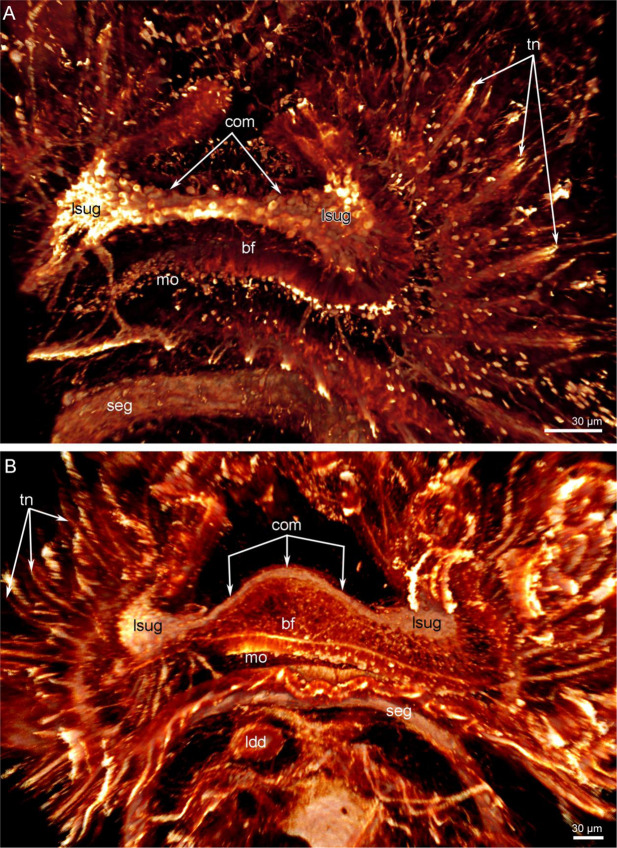
Supraenteric ganglion in juveniles of *Novocrania anomala*; volume rendering. Tubulin-like immunoreactive nerve elements of the lophophore after staining for acetylated α-tubulin, (**A**) supraenteric ganglion in a juvenile with a trocholophe: two lobes and a short commissure are visible, (**B**) supraenteric ganglion in a juvenile with a schizolophe: two lobes and a long commissure are visible. Abbreviations: bf, brachial fold; com, commissure-like nerve cord; ldd, lobe of digestive diverticula; lsug, lobe of supraenteric ganglion; mo, mouth; seg, subenteric ganglion; tn, tentacular nerves.

**Figure 4:**
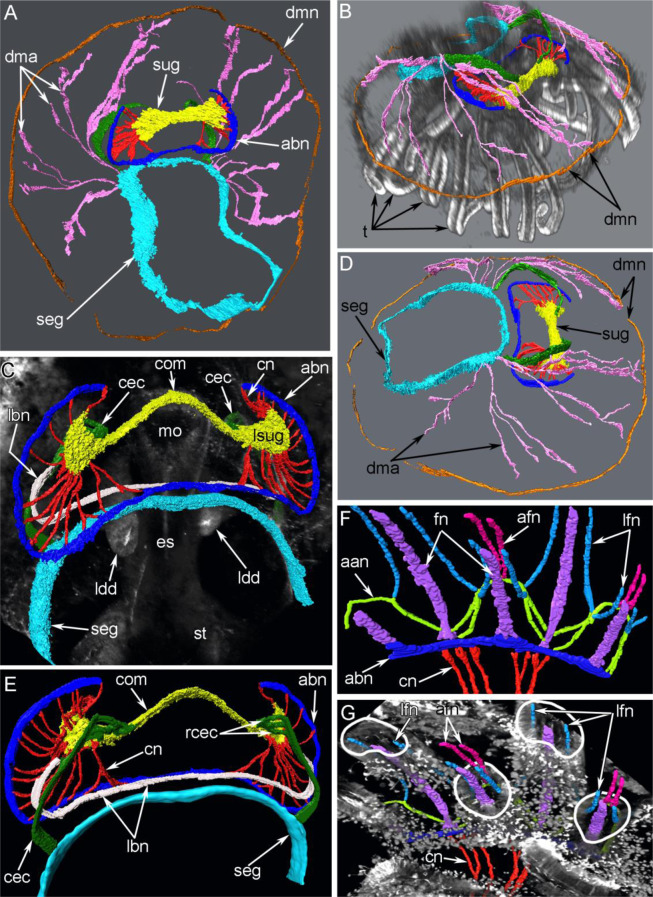
Three-dimensional reconstructions of the nervous system and innervation of the lophophore and tentacles in juveniles of *Novocrania anomala*. Juvenile with a trocholophe (**A,B,D**), and a juvenile with a schizolophe (**C,E–G**). (**A**) Whole nervous system viewed from the ventral side, (**B**) three-dimensional reconstruction and volume rendering based on staining with phalloidin, viewed from the anterior right, (**C**) the lophophoral nervous system viewed from the ventral side, (**D**) three-dimensional reconstruction of the nervous system viewed from the right, (**E**) the lophophoral nervous system viewed from the dorsal side, (**F**) innervations of tentacles, (**G**) three-dimensional reconstruction of tentacular nerves with the volume rendering based on staining for acetylated α-tubulin. The solid white line indicates the shape of transversal sections of tentacles. Abbreviations: aan, additional accessory nerve; abn, accessory brachial nerve; afn, abfrontal tentacular nerve; cec, circumenteric dorsal connective; cn, cross nerve; com, commissure-like nerve cord; dma, dorsal radial mantle nerves; dmn, dorsal marginal mantle nerve; es, esophagus; fn, frontal tentacular nerve; lbn, lower brachial nerve; ldd, lobe of digestive diverticula; lfn, laterofrontal tentacular nerve; lsug, lobe of supraenteric ganglion; mo, mouth; rcec, roots of circumenteric dorsal connective; seg, subenteric ganglion; st, stomach; sug, supraenteric ganglion; t, tentacle.

**Figure 5:**
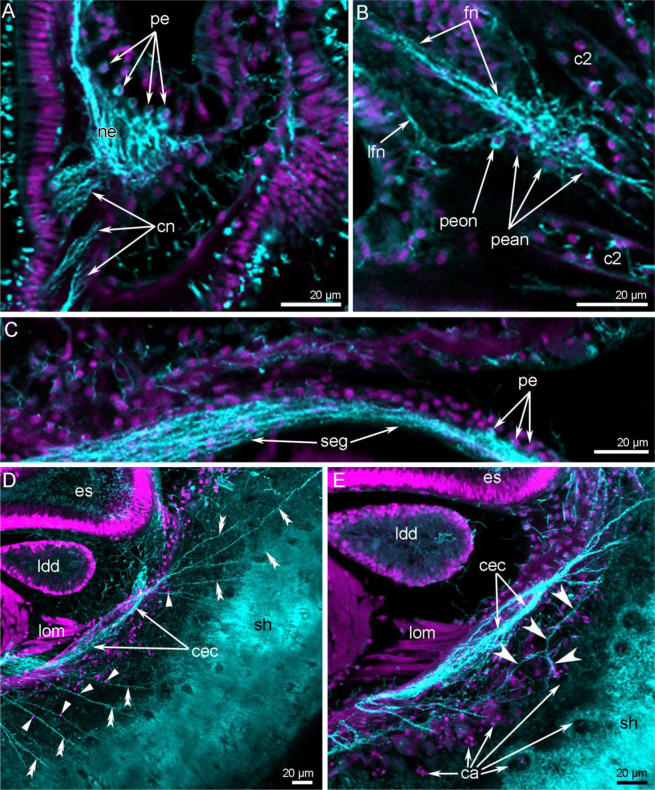
Details of the nervous system in juveniles of *Novocrania anomala*. Z-projections after double staining for acetylated α-tubulin (cyan) and DAPI (magenta). (**A**) A lobe of a supraenteric ganglion: numerous perikarya and large neuropil are visible, (**B**) perikarya of oblique nerves and accessory brachial nerve at the base of tentacle, (**C**) anterior portion of a subenteric ganglion; (**D**) dorsal radial mantle nerves (double arrowheads) and their perikarya (straight arrowheads); (**E**) thin mantle nerves (concave arrowheads), which innervate the shell caeca. Abbreviations: ca, caeca of the shell; cec, circumenteric dorsal connective; cn, cross nerve; es, esophagus; fn, frontal tentacular nerve; ldd, lobe of digestive diverticula; lfn, laterofrontal tentacular nerve; lom, lophophoral muscles; ne, neuropil; pe, perikarya; pean, perikarya of accessory brachial nerve; peon, perikarya of oblique nerves; sh, shell.

In juveniles with a trocholophe or with a schizolophe, the most prominent part of the nervous system is a large supraenteric ganglion (Figs. 3A,B and 4A–E). This ganglion is bilobed and consists of many cells that give rise to numerous neurites. These form a large neuropil and the commissure-like nerve cord between the two lobes (Fig. 5A). In juveniles with a trocholophe, the commissure-like nerve cord is very short and contains numerous perikarya, whereas in juveniles with a schizolophe, the commissure-like nerve cord is longer and contains fewer perikarya (Fig. 3A,B). In both cases, the commissure contains perikarya and should be regarded as part of the ganglion.

**Figure 6:**
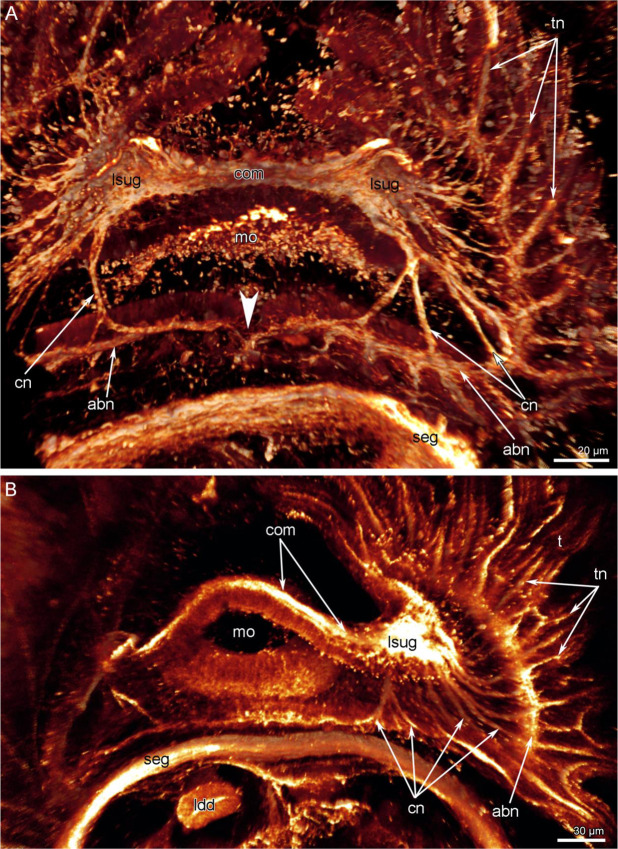
Innervation of the lophophore in juveniles of *Novocrania anomala*; volume renderings. Tubulin-like immunoreactive nerve elements of the lophophore after staining for acetylated α-tubulin. (**A**) Right arm of a trocholophe lophophore: the supraenterig ganglion gives rise to many cross nerves. The point of connection of left and right halves of the accessory brachial nerve is indicated by an arrowhead. (**B**) Right arm of a schizolophe lophophore. Abbreviations: abn, accessory brachial nerve; cn, cross nerve; com, commissure-like nerve cord; ldd, lobe of digestive diverticula; lsug, lobe of the supraenteric ganglion; mo, mouth; seg, subenteric ganglion; t, tentacle; tn, tentacular nerves.

The supraenteric ganglion gives rise to several nerve tracts: cross (transversal) nerves and circumenteric dorsal connectives.(Fig. 4A–E). In juveniles with a trocholophe or with a schizolophe, cross (transversal) nerves originate from the two lobes of the supraenteric ganglion and extend to the accessory brachial nerve (Fig. 6A,B). The most posterior cross nerves are the largest and form the circumoral connectives (Fig. 6A).

**Figure 7:**
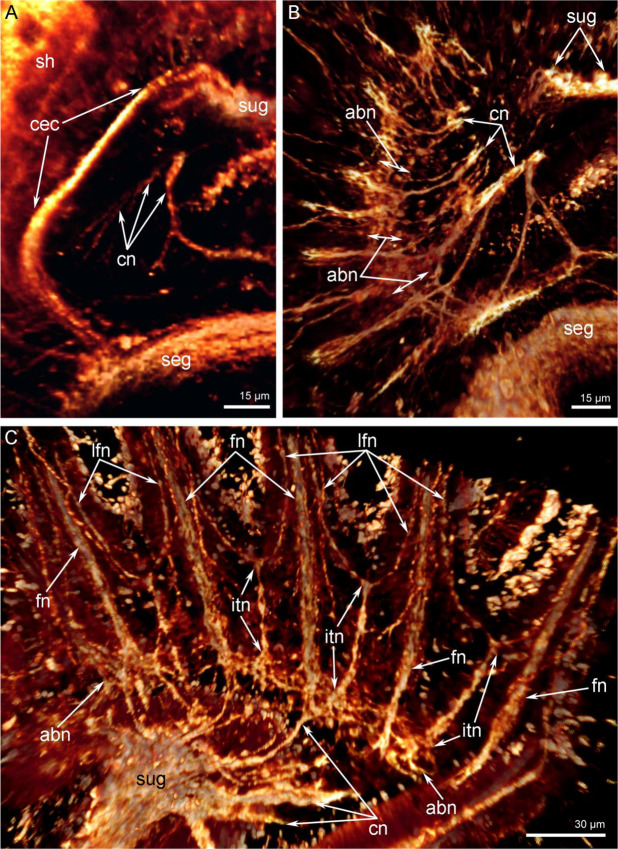
Some nerve elements of the lophophore and tentacles in juveniles of *Novocrania anomala*; volume renderings. Tubulin-like immunoreactive nerve elements of the lophophore after staining for acetylated α-tubulin. (**A**) Right circumenteric dorsal connective viewed from the dorsal side. (**B**) Cross nerves and accessory brachial nerve in a juvenile with a trocholophe. (**C**) The innervations of tentacles in a juvenile with a trocholophe: the alternation of intertentacular and frontal nerves is evident. Abbreviations: abn, accessory brachial nerve; cec, circumenteric dorsal connective; cn, cross nerve; fn, frontal tentacular nerve; itn, intertentacular nerve; lfn, laterofrontal tentacular nerve; seg, subenteric ganglion; sh, shell; sug, supraenteric ganglion.

The circumenteric dorsal connectives extend via three short roots from the lobes of the supraenteric ganglion to the subenteric ganglion (Figs. 4E and 7A). The accessory brachial nerve looks like a thick net of numerous thin neurites at the base of the tentacles (Fig. 7B). In juveniles with a trocholophe, the accessory brachial nerve seems to be composed of two halves (left and right), which are connected mid-ventrally to the mouth (Fig. 6A). The accessory brachial nerve follows the shape of the lophophore and gives rise to the tentacle nerves.

A large aggregation of perikarya that does not resemble a ganglion is located below the mouth: it is the thick circumvisceral nerve (Fig. 4A,B). It extends around the middle part of the dorsal side of the body to the posterior end and consists of many neurites and perikarya (Fig. 5C). The presence of perikarya indicates that the circumvisceral nerve should be regarded as the subenteric ganglion. It gives rise to numerous bundles of dorsal–radial mantle neurites. These neurite bundles usually originate from two dorsolateral regions of the subenteric ganglion (Fig. 4A,D). Most of the mantle neurite bundles are radial: they extend from the subenteric neurites to the edge of the mantle, where the dorsal–marginal mantle nerve runs (Figs. 4A,B,D, 5D). There are thick and thin types of the mantle neurite bundles, both of which are associated with perikarya (Fig. 5D). Some of the mantle neurite bundles branch and give rise to very thin neurites, which form a dense net in the mantle. Most of the thin neurites are associated with caeca, which extend into the shell (Fig. 5E).

Juveniles with schizolophes have a lower brachial nerve (Fig. 4C,E). It extends between two circumenteric dorsal connectives and is located below the mouth.

### Innervation of tentacles

**Figure 8:**
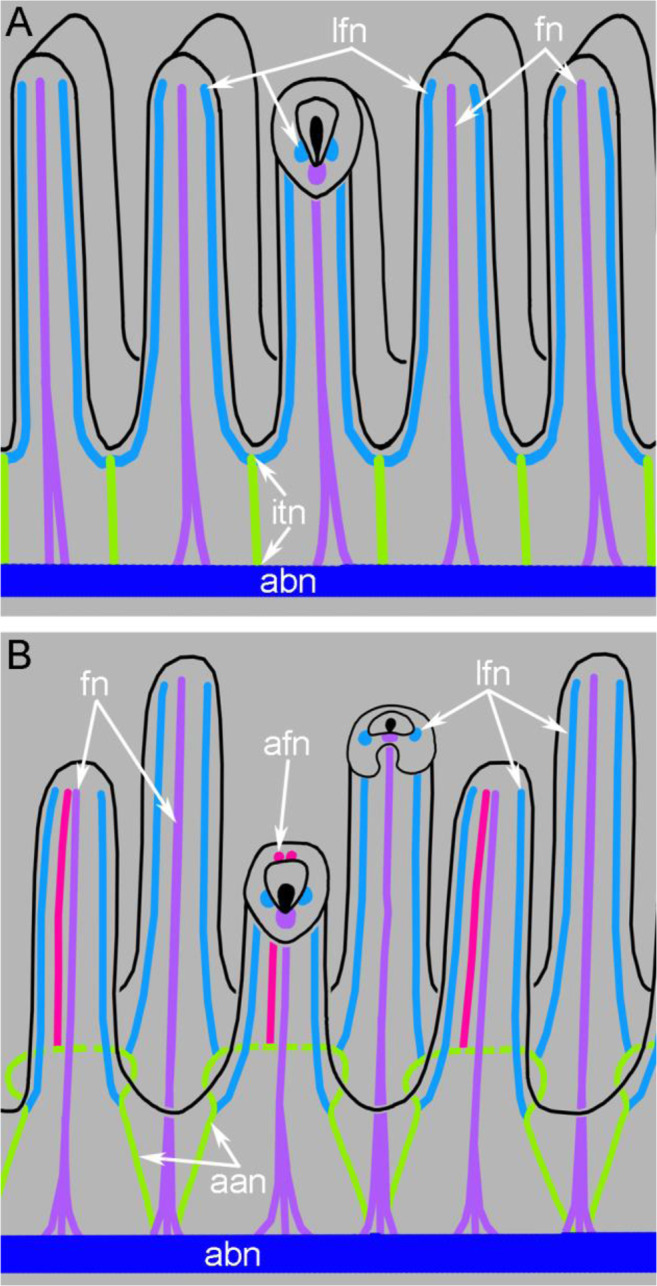
Schemes of the tentacle innervation in juveniles of *Novocrania anomala*. (**A**) Innervations of tentacles in juveniles with a trocholophe. (**B**) Innervations of tentacles in juveniles with a schizolophe. Abbreviations: abn, accessory brachial nerve; afn, abfrontal tentacular nerve; fn, frontal tentacular nerve; itn, intertentacular nerve; lfn, laterofrontal tentacular nerve.

During ontogenesis of juveniles the innervation of the tentacles changes and occurs in different ways. This difference is correlated with the reorganization of the tentacle apparatus. In juveniles with a trocholophe, the innervation of tentacles is completely supplied by an accessory brachial nerve (Fig. 7C). Two groups of neurite bundles, frontal and intertentacular, extend from the accessory brachial nerve (Fig. 7C, 8A). Frontal neurite bundles penetrate into each tentacle and extend along the frontal zone of the tentacle; i.e. these are the frontal tentacular nerves (Fig. 7C). The intertentacular nerves extend from the accessory brachial nerve to the base between adjacent tentacles, where they branch and form two neurite bundles that penetrate into adjacent tentacles and give rise to the laterofrontal tentacular nerves (Fig. 8A).

**Figure 9:**
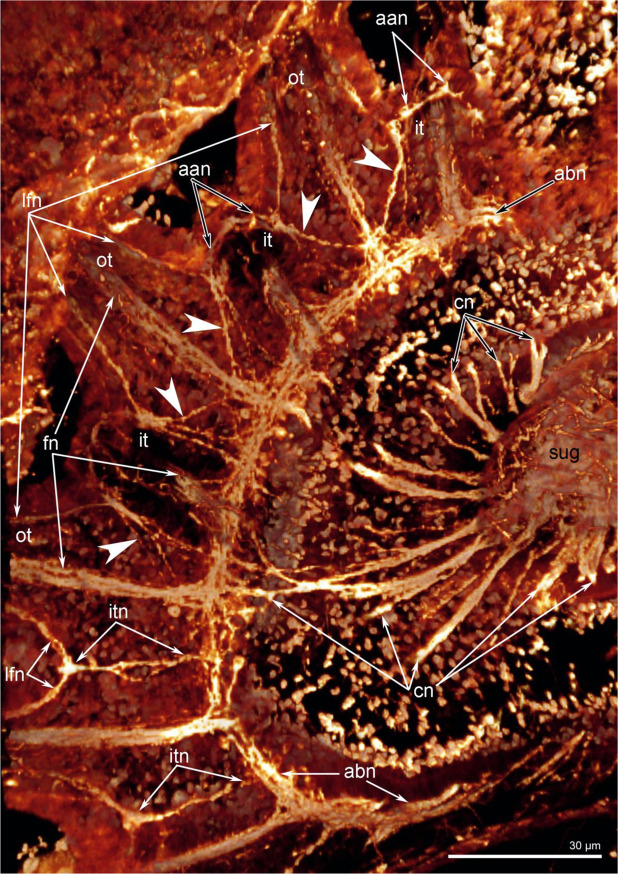
Innervation of tentacles in juveniles of *Novocrania anomala* with a schizolophe; volume rendering. Tubulin-like immunoreactive nerve elements of the lophophore after staining for acetylated α-tubulin. In the lower left corner, the innervations of one row of tentacles is visible: there is an alternation of intertentacular and frontal tentacular nerves. On the left, two rows of tentacles are visible; the intertentacular nerves are absent, and the additional accessory nerves are present. Oblique nerves of the additional accessory brachial nerve are indicated by arrowheads. Abbreviations: aan, additional accessory nerve; abn, accessory brachial nerve; afn, abfrontal tentacular nerve; cn, cross nerve, fn, frontal tentacular nerve; it, inner tentacle; itn, intertentacular nerve; lfn, laterofrontal tentacular nerve; ot, outer tentacle.

In juveniles with a schizolophe, the innervation described in the previous paragraph applies only to the oral tentacles (Fig. 9). Along the lateral sides of the lophophore, where the tentacles of the outer row appear, the innervation of tentacles is more complicated than described for earlier juvenile stages that have only one row of tentacles (Figs. 4F, 8B and 9). In juveniles with two rows of tentacles, frontal neurite bundles penetrate into the outer and inner tentacles. The frontal neurite bundles of the outer tentacles branch at their base. They give rise to short, oblique neurites, which are directed to the left and right and which extend to the base of the abfrontal side of adjacent inner tentacles (Fig. 5B). At the abfrontal base of the inner tentacles, oblique neurites with different orientations fuse and form a kind of additional accessory nerve (Figs. 8B, 9). The additional accessory nerve gives rise to the laterofrontal nerves of the inner and outer tentacles and to the abfrontal nerves of the inner tentacles (Fig. 4F). Thus, each outer tentacle is innervated by one frontal and two laterofrontal neurite bundles, whereas each inner tentacle is innervated by one frontal, two laterofrontal, and two abfrontal neurite bundles (Fig. 4G).

## Discussion

### Morphology of the lophophore and tentacles in brachiopods

In extant and extinct brachiopods, there are at least nine different types of lophophores^10^. Two types, the taxolophe and the trocholophe, may be distinguished in the postembryonic development of all species studied to date^22^. At the same time, some brachiopods have a trocholophe throughout all ontogenetic stages^23,24^. The taxolophe and trocholophe types of lophophore only have one row of tentacles and only one small coelomic canal. The schizolophe type of lophophore has two rows of tentacles and a large and a small coelomic canal. The morphology of the schizolophe is similar to that of the horseshoe-shaped lophophore of phoronids and phylactolaemate bryozoans. In schizolophes, one row of tentacles is retained below the mouth. The nomenclature used for these tentacles is inconsistent: they are described as inner tentacles in *N. anomala*^25^ but as outer tentacles in “articulated brachiopods”^26^.

**Figure 10:**
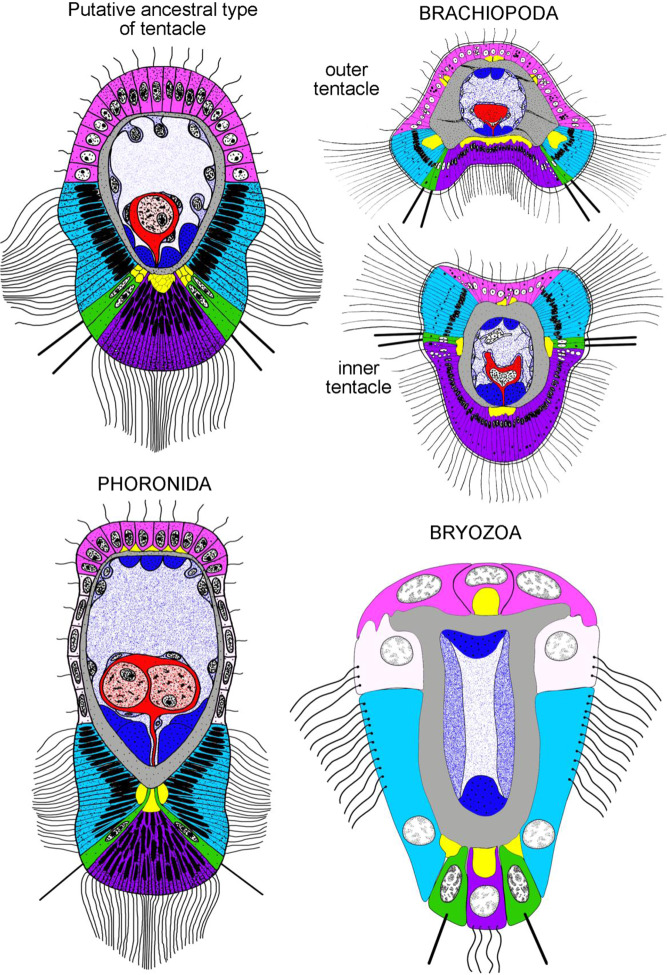
Schemes of organization of tentacles in the hypothetical ancestor of the lophophorates and in different groups of recent lophophorates. Schemes are based on (with changes): Brachiopoda^11,12^, Phoronida^21,48–52^, Bryozoa^31–36,47,53^. In all schemes, the frontal zone is facing down, the abfrontal zone is at the top. Similar zones are marked in the same color. Color table: yellow – tentacular nerves, cyan – lateral zone, green – laterofronal zone, pink – abfrontal zone, purple – frontal zone, red – tentacular blood vessel, blue – tentacular longitudinal muscles.

According to the data obtained in the present study, the oral tentacles (which are located below the mouth) may be described as ‘phoronid-like’ tentacles, which lack the specific characteristics that are typical for the inner and outer tentacles of brachiopods (Fig. 10). These specific characteristics are ridges in the inner tentacles and a groove in the outer tentacles^22,27^. ‘Phoronid-like’ tentacles may be regarded as tentacles of the primitive type, which might be ancestral for all lophophorates (Fig. 10). Although this type of tentacles may be considered to be ‘unspecialized’ in comparison with brachiopod and bryozoans tentacles, they are undoubtedly specialized in comparison with tentacles of some oweniids^28^. The specialization of phoronid-like tentacles is expressed in the co-localization of ciliated zones, nerve tracts, and muscles^21^.

### Anatomy of the nervous system

Anatomy of the nervous system of juveniles of *N. anomala* is similar to that in adults^6^, but exhibits several main differences. First, adults of *N. anomala* lack a supraenteric ganglion^6^. At the same time, juveniles with trocholophes or schizolophes have a well-developed ganglion dorsally to the mouth. This ganglion is bilobed, and the two lobes are connected by a commissure-like nerve cord. Because this commissure extends with development, the two lobes of the supraenteric ganglion move away from each other. In late juvenile stages and in adults, this commissure probably becomes the main brachial nerve, whereas the two lobes of the ganglion are probably located at terminal end of each brachial arm. These possibilities should be tested by the examination of juvenile development and organization of the lophophore nervous system in adults of other brachiopods. The transformation of the supraenteric ganglion into the main brachial nerve indicates that research is needed on the development and morphology of the main brachial nerve in brachiopods that are known to have a prominent supreaenteric ganglion.

The second difference is the presence of additional accessory nerve in juveniles (this study) and its absence in adults^6^. This nerve is discovered in late juveniles, which have two rows of tentacles. Moreover, the additional accessory nerve appears as a result of the split of intertentacular nerves, which retain only between oral tentacles.

The second accessory brachial nerve was first described as a neurite bundle that extends between the bases of two rows of tentacles in *Hemithiris psittacea*^12^. In *H. psittacea*, the second accessory nerve surrounds the frontal sides of both the inner and outer rows of tentacles^12^. In juveniles of *N. anomala*, in contrast, the additional accessory nerve surrounds the abfrontal sides of only the inner tentacles. Because of its location, the second accessory nerve of *H. psittacea* cannot be regarded as the homologue of the additional accessory nerve of *N. anomala*. This inference, however, should be supported by data from *N. anomala* adults. Based on the available data, the presence of additional nerves in *N. anomala* juveniles with a schizolophe and in *H. psittacea* adults may be regarded as an increase in complexity of the innervation of tentacles. In late juveniles of *N. anomala*, this modification is expressed in the appearance of paired abfrontal nerves in the inner tentacles. At the same time, the outer tentacles of late juveniles of *N. anomala* lack abfrontal nerves. It would be useful to examine the innervation of tentacles in *N. anomala* adults to determine whether the outer tentacles have abfrontal nerves and, if they do, their roots.

### Innervation of tentacles

**Figure 11:**
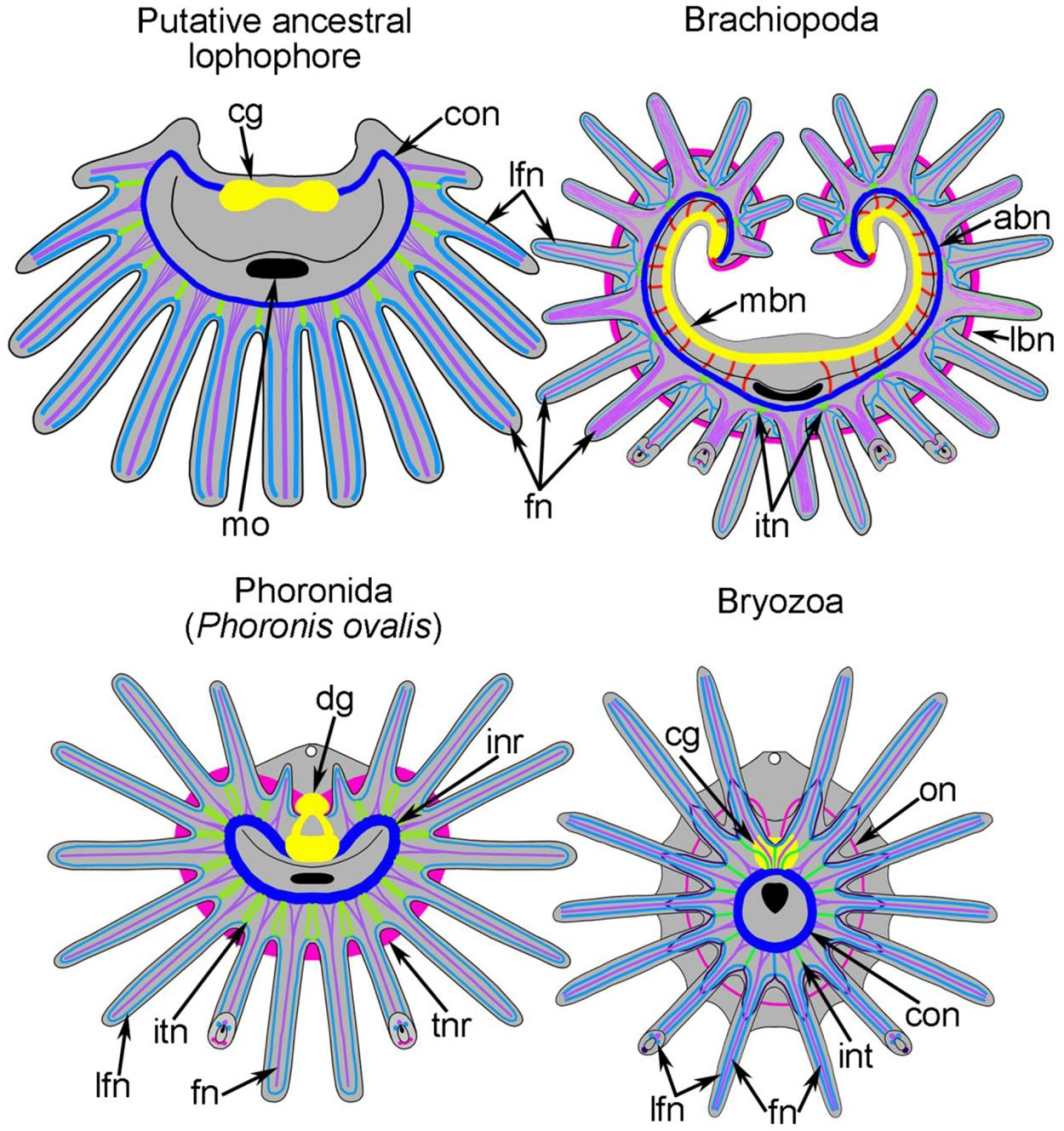
Schemes of organization of the lophophoral nervous system in hypothetical last common lophophorates ancestor and in different groups of recent lophophorates. Schemes are based on (with changes): Brachiopoda^11,12^, Phoronida^21,48–55^, Bryozoa^31–36,47,54^. In all schemes, the oral side is facing down, the anal side is at the top. Nerve elements, which are supposed to be homologous, are done in the same color. Abbreviations: abn, accessory brachial nerve; cg, cerebral ganglion; dg, dorsal ganglion; fn, frontal tentacular nerve; inr, inner nerve ring; itn, intertentacular nerve; lbn, lower brachial nerve; lfn, laterofrontal tentacular nerve; mbn, main brachial nerve; on, outer nerve ring; tnr, tentacular nerve ring.

Innervation of brachiopod tentacles is described for several species^11,12,29,30^. The current findings indicate that the innervation of *N. anomala* tentacles differs substantially in juveniles with a trocholophe and juveniles with the schizolophe (Fig. 8). This difference is related to the presence of one row of tentacles with trocholophes vs. two rows of tentacles for schizolophes. In the trocholophe, the innervation pattern is simple: there are only frontal and intertentacular nerves, which extend from the accessory brachial nerve. The same pattern may be distinguished in different groups of bryozoans^31–36^ and in *Phoronis ovalis*^21^, in which the circumoral nerve ring gives rise to the frontal and intertentacular nerves (Figs. 10, 11). This pattern leads to the presence of only three neurite bundles in each tentacle: one frontal and two laterofrontal. The pattern becomes more complicated when additional tentacular nerves appear. These nerves may originate from frontal and laterofrontal bundles as in bryozoans, or from an outer nerve ring as in phoronids.

In adult brachiopods studied to date by transmission electron microscopy, antibody staining, and confocal laser scanning microscopy, the accessory brachial nerve (in *Lingula anatina*) or the main brachial nerve (in *Hemithiris psittacea*) gives rise only to frontal neurite bundles, which then branch to form the nerves that extend into the outer and inner tentacles^11,12^. Thus, in adult brachiopods, the brachial nerves give rise to the frontal neurite bundles but not to the intertentacular neurite bundles that are present in those juveniles of *N. anomala* that have a single row of tentacles. The current results suggest that the “adult” pattern of tentacle innervation might result from the formation of the second row of tentacles, which originate exactly in the location of the intertentacular nerves. The appearance of a second row of tentacles leads to the disappearance of the integral intertentacular nerves, which split into several neurite bundles that form the additional accessory brachial nerve.

### Evolution of the lophophore and tentacles in the lophophorates

The monophyly of the lophophorates recently gained support from transcriptomic, phylogenomic, and morphological studies^11,20,21,34,35,37–40^. Determining how the ancestral lophophore was organized will increase our understanding of the evolution of recent lophophorates and the evolution of many extinct “tentaculates” including the enigmatic hyoliths and other groups^41–43^. The lophophore is a specialized organ, with a structure that is adapted to collect food particles from the water column. As previously suggested^44^, the ancestral lophophore had a simple morphology and was probably ovoid or crescent-shaped, bore one row of tentacles, and contained a single coelomic cavity. According to the data concerning the organization of the lophophoral nervous system of *N. anomala* (this study) and of other lophophorates, the nervous system of the ancestral lophophore may have consisted of only a few nerve elements: the cerebral ganglion, the circumoral nerve ring, and tentacular nerves (Fig. 11). In extant lophophorates, the cerebral ganglion corresponds to a dorsal ganglion in phoronids, a supraenteric ganglion in brachiopods, and a cerebral ganglion in bryozoans. The circumoral nerve ring corresponds to an inner nerve ring in phoronids, an accessory brachial nerve in brachiopods, and a circumoral nerve ring in bryozoans. In each tentacle of the last common lophophorate ancestor, there were at least three groups of nerves: one frontal and two laterofrontal. Such an arrangement is correlated with the zones with the highest density of cilia, i.e., the frontal and laterofrontal zones. The presence of intertentacular nerves and the innervation of adjacent tentacles from the one intertentaculate nerve are important. This innervation allows the coordination of the ciliary activity of laterofrontal zones of adjacent tentacles, whose cilia function as a mesh to capture food particles^22,45,46^. The appearance of the second nerve center and the second nerve ring^21^, which extends at the base of tentacles, may be regarded as a way to innervate different zones of tentacles to improve their function.

Among lophophorates, phoronids have the least specialized tentacles. In brachiopods, the specialization is expressed in the formation of two types of tentacles with different morphologies: inner and outer tentacles^22,27^. In bryozoans, the specialization of tentacles is related to their miniaturization: in transversal sections of bryozoan tentacles, each zone is represented by a few cells or even a single cell^47^. The presence of ‘phoronid-like’ tentacles in brachiopod ontogeny, in all adult brachiopods (as oral tentacles), and in phoronids suggests that this type of tentacle is ancestral for all lophophorates.

### Larval and juvenile nervous systems

*Novocrania anomala* has lecitotrophic larvae that undergo a metamorphosis resulting in the formation of the shell and the lophophore^13^. The larval nervous system changes into the juvenile nervous system with the disappearance of the apical organ and the formation of new nerve cords^16^. Two lateral neurite bundles are well-developed in the ventral side of the apical lobe in 5-day-old juveniles^16^. Based on their position (along the lateral sides of the body), these “ventral neurite bundles”^16^ may be compared with the circumenteric dorsal connectives, which were found in juveniles in the current study. The ventral or dorsal position is impossible to be determined without a whole-3D reconstruction of the nervous system and the body. According to Altenburger and Wanninger^16^, supraenteric and subenteric ganglioa originate as commissures between two “ventral neurite bundles”. Previous data and the current findings suggests that the anterior commissure gives rise to the supraenteric ganglion, the median commissure, the posterior commissure, and the “ventral neurite bundles”; the latter elongates in a posterior direction and forms the subenteric ganglion, which is the large circumvisceral nerve.

According to the current findings, the radial mantle nerves in *N. anomala* extend from two dorsolateral points of the subenteric ganglion to the dorsal mantle nerve, which runs along the mantle margin. In adults, the mantle nerves also originate from the two dorsolateral points of the subenteric ganglion^6^. According to Altenburger and Wanninger^16^, “serially arranged mantle neurites” extend from anterior portions of the “ventral neurite bundles” and also from the anterior commissure. It seems possible that Altenburger and Wanninger^16^ may have regarded some of the lophophoral nerves as mantle nerves.

Planktotrophic larvae of linguliform brachiopods have lophophores with tentacles which are innervated from ventral and dorsal lophophoral nerve rings^55,56^. The ventral nerve ring gives rise to the frontal tentacular nerves; the dorsal nerve ring gives rise to the abfrontal tentacular nerves^54^. Researchers have generally concluded that linguliform brachiopods do not undergo any catastrophic changes in morphology during metamorphosis^57,58^. Thus, the larval trocholophe is inherited by the juvenile and develops into the schizolophe. Although the transformation of the nervous system has not been studied, we can infer that the larval nervous system is retained by the juvenile. An exception concerns the median tentacle, which has serotonin-like immunoreactive perikarya that disappear during metamorphosis. Based on their contribution to the innervation of tentacles, the ventral and dorsal lophophoral nerve rings of the larva may develop into the accessory and lower brachial nerves of the juvenile. The main brachial nerve and the subenteric ganglion, which have been described in adult *Lingula anatina*^7,11^, probably develop *de novo* in juveniles. This suggestion raises the question how the supraenteric ganglion and the main brachial nerve develop and are organized in other adult brachiopods, which are documented to have a prominent supraenteric ganglion.

## Materials and methods

### Sampling of animals and light microscopy

In the North Sea (Storingavika Bay) in May 2019, a drag net was used to collect stones and adhering juveniles of *N. anomala*. The juveniles were carefully separated from the stones, except that the ventral valves remained attached to the stones. Live animals were photographed from the ventral side using a Leica M165C (Leica, Germany) stereomicroscope equipped with a Leica DFC420 digital camera.

### Immunocytochemistry

In general, for this part of work I have used the protocol, which was previously efficient in the study of the lophophore nervous system in phoronids^21^ and brachiopods^11,12^. Whole juveniles were fixed in a 4% paraformaldehyde solution in filtered sea water and washed in phosphate buffer (pH 7.4) (Fisher Scientific, Pittsburgh, PA, USA) with Triton X-100 (1%) (Fisher Scientific) (PBT) for a total of 24 h. Nonspecific binding sites were blocked with 12% normal donkey serum (Jackson ImmunoResearch, Newmarket, Suffolk, UK) in PBT overnight at 4 °C. The specimens were then transferred into primary antibody anti-α-Tubulin-mouse (1:600) (ImmunoStar, Hudson, WI, USA) in PBT, and incubated for 24 h at 4 °C with rotation. Specimens were washed in PBT and were then exposed to the secondary antibody, 635-Alexa-Mouse (1:1000) (Invitrogen, Grand Island, NY, USA) in PBT, for 24 h at 4 °C. Before washing, DAPI and phalloidin (Alexa-488) were added to the vials with animals at concentration ratios of 1:300 and 1:250, respectively. After they were washed in phosphate buffer, the specimens were embedded in Murray Clear (50:50, benzyl benzoate:benzyl alcohol). Specimens were examined with a Nikon Eclipse Ti confocal microscope (Nikon, ThermoFisher Scientific, Waltham, MA). Z-projections were obtained using Image J version 1.43. Three-dimensional reconstructions and volume renderings were produced with Amira version 5.2.2 (Thermo Fisher Scientific, USA).

### Scanning electron microscopy (SEM)

For SEM the standard protocol was used: specimens were fixed in 2.5% glutaraldehyde in 0.2 M cacodylate buffer overnight. The specimens were then washed in 0.2 M cacodylate buffer and postfixed in 1% osmium tetroxide in the same buffer for 3 h at room temperature. The specimens were dehydrated in ethanol and acetone, critical point dried, and then sputter coated with platinum-palladium. Specimens were examined with a Jeol JSM scanning electron microscope (JEOL Ltd., Tokyo, Japan).

### Terminology

The terminology, which is used in this report, is in line with the traditional terminology that was suggested in first papers devoted to the brachiopod nervous system^4–7,58^. This traditional terminology is often inconsistent with the recent knowledge on organization of the nervous system^59^. Thus, so-called brachiopod ganglia are not true ganglia; they are formed by neurioepithelia^60^. In order to escape the misunderstanding in description of the brachiopod nervous system, I have used the traditional terminology.

### Ethics statement

The field sampling did not involve endangered or protected species. The use of brachiopods in the laboratory does not raise any ethical issues.

## Data Availability

The data sets analyzed during this study are available from the author upon request
